# Cholinergic Pathway SNPs and Postural Control in 477 Older Adults

**DOI:** 10.3389/fnagi.2018.00260

**Published:** 2018-09-04

**Authors:** Carina Arnold, Claudia Schulte, Mariana Moscovich, Ulrike Sünkel, Laura Zaunbrecher, Florian Metzger, Thomas Gasser, Gerhard W. Eschweiler, Ann-Kathrin Hauser, Daniela Berg, Walter Maetzler

**Affiliations:** ^1^Department of Neurodegenerative Diseases, Center of Neurology, Hertie Institute for Clinical Brain Research, University of Tübingen, Tübingen, Germany; ^2^German Center for Neurodegenerative Diseases, Tübingen, Germany; ^3^Department of Neurology, University of Kiel, Kiel, Germany; ^4^Geriatric Center at the University Hospital of Tübingen, Tübingen, Germany; ^5^Department of Psychiatry and Psychotherapy, University Hospital of Tübingen, Tübingen, Germany

**Keywords:** acetylcholine, balance control, genetic markers, neurodegeneration, single nucleotide polymorphisms (SNPs)

## Abstract

**Objective:** To determine whether single nucleotide polymorphisms (SNPs) of the cholinergic system and quantitative parameters of postural control are associated in healthy older adults. This is a cross-sectional analysis from the TREND study.

**Methods:** All participants performed a static postural control task for 30 s on a foam pad in semitandem stance and eyes closed. We analyzed mean power frequency (MPF), area, acceleration, jerk, and velocity from a mobile sensor worn at the lower back using a validated algorithm. Genotypes of four SNPs in genes involved in the cholinergic system (*SLC5A7, CHAT, BCHE, CHRNA4*) were extracted from the NeuroX chip. All participants present a normal neurological examination and a Minimental state examination score >24.

**Results:** Four hundred and seventy seven participants were included. Mean age was 69 years, 41% were female. One SNP of the cholinergic pathway was significantly associated with a quantitative postural control parameter. The minor allele of rs6542746 in *SLC5A7* was associated with lower MPF (4.04 vs. 4.22 Hz; *p* = 3.91 × 10^-4^). Moreover, the following associations showed trends toward significance: minor allele of rs6542746 in *SLC5A7* with higher anteroposterior acceleration (318 vs. 287 mG; *p* = 0.005), and minor allele of rs3810950 in *CHAT* with higher mediolateral acceleration [1.77 vs. 1.65 log(mG); *p* = 0.03] and velocity [1.83 vs. 1.74 log(mm/s); *p* = 0.019]. Intraindividual occurrence of rs6542746 and rs3810950 minor alleles was dose-dependently related with lower MPF (*p* = 0.004).

**Conclusion:** This observational study suggests an influence of SNPs of the cholinergic pathway on postural control in older adults.

## Introduction

Postural instability is a common and disabling feature of older adults. One in three adults aged 65 and older will fall at least once a year (Frenklach et al., 2009). Falls result frequently in injuries such as fractures, and lead to fear of future falls accompanied by decreased mobility and decreased quality of life (Adkin et al., 2003; Michalowska et al., 2005). Thus, postural instability and falls represent a serious health problem (Tinetti and Williams, 1997; Manckoundia et al., 2008).

A fall is most often a consequence of a postural control error, and repeated occurrence of falls indicate a postural control deficit. In recent years, evidence has become consistent that degeneration of cholinergic neurons in the pedunculopontine nucleus (PPN) -one of the three main sources of acetylcholine supply in the brain- plays a central role in impaired postural control (Lee et al., 2000; Bohnen et al., 2009; Kucinski and Sarter, 2015). The cholinergic pars compacta of the PPN supplies the majority of cholinergic input to the thalamus and has reciprocal connections with the basal ganglia nuclei, particularly the substantia nigra pars compacta, globus pallidus and subthalamic nucleus (Lee et al., 2000; Pahapill and Lozano, 2000; Yarnall et al., 2011). Supporting the cholinergic relevance in postural control, several studies (Bohnen et al., 2009; Karachi et al., 2010; Rochester et al., 2012; Muller et al., 2013) indicate that degeneration of the cholinergic part of the PPN plays a central role in impaired postural control and gait dysfunction in Parkinson disease (PD) and other degenerative diseases. Moreover, studies demonstrated improvement in falls rate and postural control parameters by treatment with anticholinesterase inhibitors (Chung et al., 2010; Henderson et al., 2016). Interestingly, we did not find data about degeneration of this system in association with age and postural control in the “healthy” older population.

The cholinergic system is complex and comprises various enzymes, transporters, and receptors that interact with, or release the neurotransmitter acetylcholine during the propagation of a nerve impulse. Some single nucleotide polymorphisms (SNPs) of genes from this cholinergic system have been suggested to have an influence on the occurrence of specific neurodegenerative diseases (Ozturk et al., 2006). Examples are SNPs in the sodium ion- and chloride ion-dependent choline transporter (*SLC5A7)* rs6542746, the choline *O*-acetyltransferase *(CHAT)* rs3810950, the butyrylcholinesterase (*BCHE)* rs1803274 gene and the cholinergic receptor nicotinic alpha 4 subunit *(CHRNA4)* rs2236196. *SLC5A7* is the gene encoding choline transporter (ChT). The high-affinity ChT plays an essential role in the control of ACh synthesis. A specific impairment in cognitive control associated with a variant of *SLC5A7* has been reported (Berry et al., 2014). However, functional consequences of the analyzed variant are not yet investigated. *CHAT* is the gene encoding choline acetyltransferase (ChAT), the key enzyme responsible for the synthesis of the neurotransmitter acetylcholine. ChAT activity decreases in the brains of patients with PD (Ruberg et al., 1982; Lange et al., 1993), a disease closely associated with postural control deficits. The AA genotype of the analyzed SNP was associated with increased risk for AD and the A allele was hypothesized to increase translation of ChAT (Kim et al., 2004). Definite functional consequences were not investigated. The *BCHE* gene encodes butyrylcholinesterase. The SNP rs1803274 is the so-called BCHE K-variant (T allele) that has been reported to lead to reduced hydrolytic activity of the gene product (Podoly et al., 2009). The mechanism of action is a prolonged maintenance of acetylcholine (Ach) at the synaptic cleft due to an increase in concentration. *CHRNA4* is the gene encoding the cholinergic receptor nicotinic alpha 4 subunit in nicotinic *α*4*β*2 receptors (Yarnall et al., 2011). SNP variation seems associated with various cognitive and behavioral changes including schizophrenia (Reinvang et al., 2010; Greenwood et al., 2012; Eggert et al., 2015) although a recent meta-analysis does not support a highly relevant role of this SNP in schizophrenia (Schizophrenia Working Group of the Psychiatric Genomics Consortium, 2014). Recent research has shown that a variation in CHRNA4 affects attentional networks of the brain (Reinvang et al., 2009) and negative emotionality in normal adults (Tsai et al., 2012). Functional consequences of the analyzed variant are not yet investigated.

Static postural control is a central feature of our postural control system (Maetzler et al., 2013b), and the decline of this ability during the process of aging has repeatedly been demonstrated (Berg et al., 1992; Woollacott, 2000; Van Ooteghem et al., 2009). Generally, the basic assumption in postural control patterns is that more sway is associated with worse postural control (Maetzler et al., 2013a). Deficits in postural control can be identified by wearable sensors, which can detect even subtle changes of sway patterns (Mancini et al., 2012b; Maetzler et al., 2013a). The following parameters are often used to describe this pattern (Rocchi et al., 2004; Maetzler et al., 2012): mean power frequency (MPF), area, acceleration, jerk and velocity (Mancini et al., 2012a,b; Park et al., 2016). These parameters can vary with age and neurodegeneration. For example, MPF has been shown to be higher in older adults (age >65) when compared to young adults (age <30) (Demura and Kitabayashi, 2006; Dewhurst et al., 2007) and to be lower in clinical and even prodromal PD (Mancini et al., 2012a; Hasmann, 2015). Area has been shown to be larger in PD patients when compared with controls (Oude Nijhuis et al., 2014), and acceleration to be larger in older adults than in young adults (Era et al., 2006; Masani et al., 2007). An increase of jerk has been associated with disturbance of postural control in neurodegenerative disorders such as PD (McVey et al., 2009; Mancini et al., 2011, 2012a). Sway velocity has been associated with age (Era et al., 2006), as well as with cognitive impairment in older adults (Deschamps et al., 2014) and PD (Sciadas et al., 2016).

This work aims to discover potential associations between SNPs of the cholinergic system and quantitative parameters of postural control in a large cohort of healthy older adults. This aim is based on the idea that genetically defined differences in the performance of postural control may serve as a not yet considered risk factor for postural control deficits and consecutive falls in vulnerable cohorts.

## Materials and Methods

### Study Participants

We investigated a cohort of 477 healthy older adults, aged 56–88 years. All individuals were evaluated between 2011 and 2012 during the second assessment of the TREND study^1^. A detailed description of the study cohort has been provided previously (Hobert et al., 2011). The study was approved by the ethical board of the Medical Faculty of the University of Tübingen (No. 90/2009BO2) and all participants provided written informed consent. Inclusion criteria for the actual analysis were normal neurological examination, Minimental state examination >24, and the ability to perform a challenging postural control task without risk of falling. Exclusion criteria were more than one of the PD risk factors, REM Sleep Behaviour Disorder (RBD), hyposmia and depression, and presence of common *LRRK2* or *GBA* mutations (G2019S rs34637584, N370S rs76763715, and L444P rs421016) (Healy et al., 2008). Pallesthesia was assessed with a Rydel-Seiffer 128 Hz tuning fork (median of vibration sensitivity values on both malleoli mediales). Diagnosis of RBD was based on the RBD Screening Questionnaire, using a cut-off value of five points (Richter et al., 2014). The diagnosis of hyposmia was based on Sniffin’ Sticks. It is a validated and commonly used tool for the assessment of olfactory function (Lotfipour et al., 2011). Depression was defined as Beck Depression Inventory value ≥14 (Ellis et al., 2009). Since the origin of all included participants is within Europe, we do not expect population differences.

### Assessment and Evaluation of Static Postural Control

**FIGURE 1:**
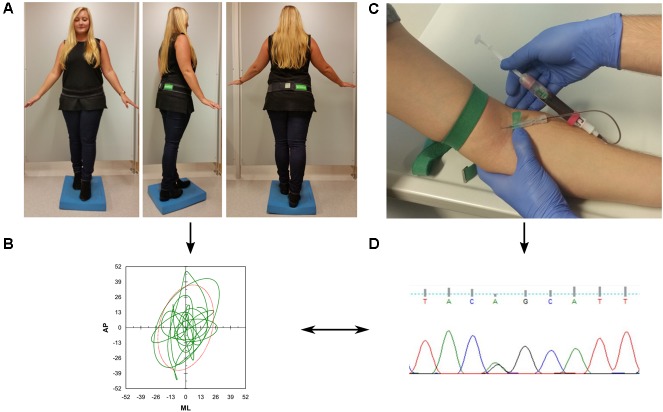
Overview of the study protocol. **(A)** Participants were asked to perform a difficult static balance task. During the assessment, they wore an inertial measurement unit (DynaPort^®^, McRoberts, Netherlands) at the lower back. **(B)** Acceleration signals were analyzed with validated algorithms provided by the company. **(C)** Collection of blood for genetic analysis **(D)**.

We used a challenging standing task to assess postural control. Participants were asked to stand for 30 s upright in a close semitandem stance with eyes closed on a foam pad (Airex balance pad, 50 cm × 41 cm × 6 cm), with a maximum distance of 5 cm between the right big toe and the left heel (**Figure 1**). In case this position was too difficult to be kept during a 10 s training period, the participant was allowed to increase the distance between the legs until the participant indicated that the position was stable enough to perform the task. Twenty-six participants were not able to perform the task in semitandem stance and were thus allowed to use parallel stance. During the task, participants wore the DynaPort^®^ sensor (McRoberts, Netherlands) at the level of the third and fourth lumbar spine segment, attached via an elastic velcro belt.

MPF, area, acceleration, jerk, and velocity were extracted. MPF is a measure for the frequency content of the signal given in Hz. It can be seen as the body’s basic oscillation (Lamoth et al., 2009). Area is a measure for a person’s needed area to maintain the body along the gravitational vertical during balancing performance. From the center of the stabilogramm, the distance to every data point is determined. Afterward, a circle with a radius covering 90% of the largest distance is constructed that is the evaluated area given in mm^2^. Acceleration describes the root mean square (RMS) mean acceleration of the compensatory movements (mG), and is presented in anteroposterior (AP) and mediolateral (ML) direction. Jerk, the time derivate of acceleration, quantifies smoothness of the compensatory movements. It is given in mG/s. Smoothness is broadly regarded as a hallmark of skilled, coordinated movement (Hogan and Sternad, 2009). Also jerk is presented in AP and ML direction. Velocity describes the mean velocity of the compensatory movements and is given in mm/s. Also this parameter is presented in the AP and ML direction.

### SNP Analysis

The genotypes of the SNPs of the *SLC5A7 (*rs6542746), *CHAT (*rs3810950), *BCHE (*rs1803274), and *CHRNA4* gene *(*rs2236196) were extracted from NeuroX array data, the dataset and procedures are described in Nalls et al. (2014). Call rate was 100% and HWE was >0.05. SNP locations and frequencies are listed in **Supplementary Table 1**.

### Statistical Analysis

Statistical analysis was performed with SPSS Statistics 22, IBM. Demographic and clinical data are presented with mean and standard deviation (SD). Quantitative postural control parameters were checked for outliers, and values ≥3 STD from the mean were excluded. Non-normally distributed parameters were log transformed before analysis (area, acceleration ML and jerk AP and ML). Comparisons of genotypes with postural control parameters were performed with a linear regression model, by including age, gender, Beck’s Depression Inventory score and pallaesthesia as potential confounders in the model.

The effect of combining SNPs SLC5A7 rs6542746 and CHAT rs3810950 was analyzed by scoring of the, respectively, rare alleles. *p*-Values were considered trends toward significance when 0.0016 < *p* < 0.05, and significant when *p* ≤ 0.0016 (four SNPs, eight postural control parameters). The reported effect sizes were calculated according to Cohen’s f^2^ score (Cohen, 1988). Data are shown in **Supplementary Table 2**.

## Results

The mean age of the cohort was 69 years, and 41.3% were women. **Table 1** provides demographic and clinical details.

**Table 1:** Means and standard deviations of relevant demographic and postural control parameters of the included cohort.

	Mean	Standard deviation
N (females)	477 (197)	–
Age (years)	69	6
BMI [weight/(height^2^)]	26	4
Height (cm)	171	9
BDI (0–63)	4	4
MMSE (0–30)	29	1
Pallesthesia (0–8)	7	2
MPF (Hz)	4.2	0.5
Area (mm^2^)	3.159	2.538
Acceleration AP (mG)	307	116
Acceleration ML (mG)	56	32
Jerk AP (mG/s)	280	455
Jerk ML (mG/s)	345	627
Velocity AP (mm/s)	64	35
Velocity ML (mm/s)	68	40

*AP, anteroposterior; BDI, Beck’s Depression Inventory; BMI, body mass index; ML, mediolateral; MMSE, Mini Mental Status Exam; MPF, mean power frequency.*

### SNP Association Analysis With Postural Control Parameters

**FIGURE 2:**
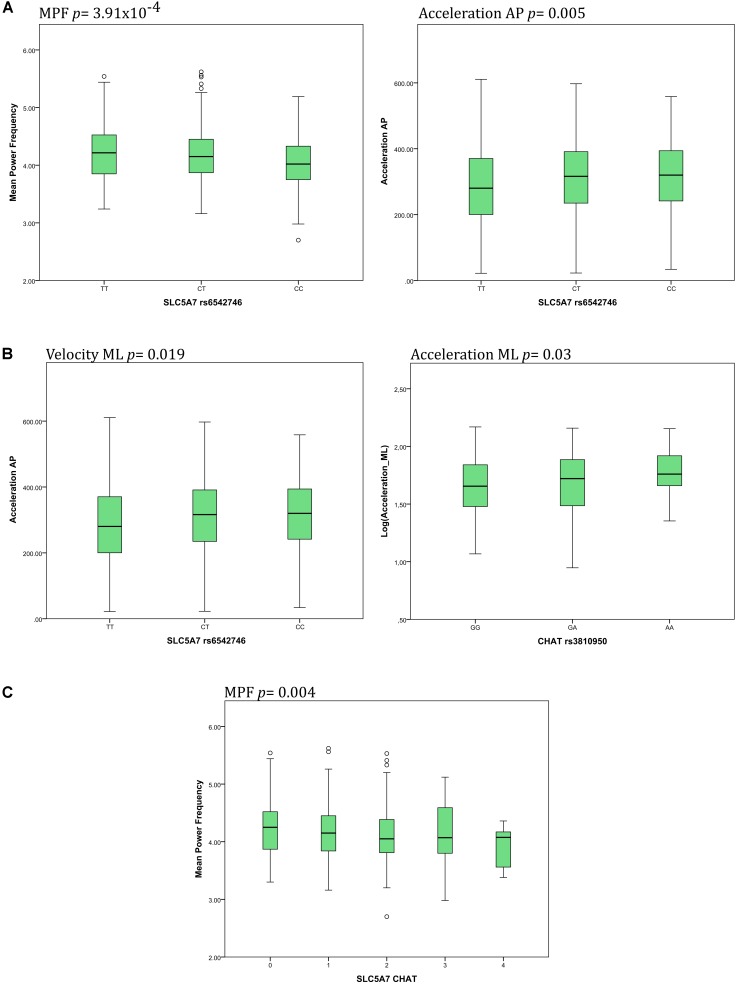
Main associations of SNPs of the cholinergic system with postural control parameters. **(A)** The *SLC5A7* SNP was associated with changes of mean power frequency (MPF) and acceleration in the anteroposterior (AP) direction. **(B)** The *CHAT* SNP was associated with changes of both velocity and acceleration in the mediolateral (ML) direction. **(C)** Increasing presence of minor alleles of both SNPs, *SLC5A7* and *CHAT*, was associated with decreasing MPF.

Details about significant results on both, a Bonferroni-corrected and uncorrected level, are presented in **Figures 2A,B**. Individuals carrying a genotype with the minor C allele in SLC5A7 rs6542746 showed lower MPF values (CC: 4.04/CT: 4.15/TT: 4.22 Hz, *p* = 3.91 × 10^-4^, *f*^2^ = 0.028), compared to the major T allele. Acceleration AP values showed a strong dose-dependent trend toward significance with higher minor allele representation (CC: 318/CT: 314/TT: 287 mG, *p* = 0.005, *f*^2^ = 0.017).

Genotypes containing the minor A allele in *CHAT* rs3810950 showed dose-dependent higher values in acceleration ML [AA: 1.77/AG: 1.70/GG: 1.65 log(mG), *p* = 0.03, *f*^2^ = 0.012], velocity ML [AA: 1.83/AG: 1.78/GG: 1.74 log(mm/s), *p* = 0.019, *f*^2^ = 0.012], jerk AP [AA: 2.28/AG: 2.20/GG: 2.15 log(mG/s), *p* = 0.048, *f*^2^ = 0.009] and velocity AP [AA: 1.80/AG: 1.75/GG: 1.74 log(mm/s), *p* = 0.064, *f*^2^ = 0.007] compared to the major G allele.

### Combination of *SLC5A7* rs6542746 and *CHAT* rs3810950

Individuals scored for the presence of the minor alleles of both SNPs in *SLC5A7* rs6542746 and in *CHAT* rs3810950 showed a dose-dependent lowering of MPF values (*p* = 0.004, *f*^2^ = 0.018, **Figure 2C**).

*BCHE* rs1803274 and *CHRNA4* rs2236196 SNPs were not significantly associated with any of the postural control parameters (data not shown).

## Discussion

During the course of aging (Heuninckx et al., 2008) and in neurological disorders such as PD (Helmich et al., 2007), postural control deficits become increasingly common in the 5th decade of life and later (Schumacher et al., 2014) and have a substantial influence on life quality of the affected (Maetzler et al., 2013b). Recent findings demonstrate that compensated postural control deficits, as they occur, e.g., during prodromal phases of neurodegenerative diseases, become detectable when this system is maximally challenged (Maetzler et al., 2012). Such a challenge can be, e.g., standing in semitandem position on a foam pad with eyes closed (Horak and Mancini, 2013). Based on consistent results of several studies (Chung et al., 2010; Karachi et al., 2010; Rochester et al., 2012; Muller et al., 2013) indicating that the cholinergic system plays a central role in postural control, genetic variation in the following four genes of the cholinergic pathway, *SLC5A7, CHAT*, *BCHE*, and *CHRNA4*, were examined in this study regarding their potential influence on postural control parameters in healthy older adults. To our best knowledge, this is the first report linking genetic variation in the cholinergic system to quantitative changes in postural control performance in a representative cohort of older adults. This research issue is particularly interesting in the light of a recent study (Richter et al., 2014), which suggests that ACHE activity in the brain is individually and locally regulated and influences specific cognitive tasks closely and specifically.

In this study, the SNP rs6542746 in the *SLC5A7* gene showed significant association with MPF and a strong trend toward significance with acceleration AP. Although some literature suggests that MPF increases with age, recent studies indicate that patients with PD in clinical (Mancini et al., 2012a) and even in their prodromal stages (Hasmann, 2015) present with reduced MPF. The reasons for this reduction of MPF in relation to neurodegenerative processes are not understood, yet. Nevertheless, our results suggest that the postural control assessment applied here has the potential to detect subtle changes of the cholinergic system beyond aging effects, and may reflect subtle changes due to, e.g., neurodegeneration. It is also possible that not an, e.g., age-related, degeneration of the cholinergic system *per se* explains our results (Ellis et al., 2009; Lotfipour et al., 2011) but rather the degeneration of the dopaminergic system or the occurrence/increase of white matter lesions (Richter et al., 2017) unmask a constitutionally “weak” cholinergic system determined by the cholinergic polymorphisms. These hypotheses have to be evaluated with longitudinal observation studies, including also the actual TREND cohort. The trend toward higher acceleration AP in the minor allele of the *SLC5A7* rs6542746 SNP also suggests worse postural control (Horak and Mancini, 2013).

Increase of acceleration in AP direction may reflect a postural control deficit that is different from an increase of acceleration in ML direction. This aspect has been nicely demonstrated in a previous experimental study (Runge et al., 1999). Eventually, increases of both AP and ML accelerations may indicate a prodromal phase of neurodegeneration (Maetzler et al., 2012).

The *CHAT* rs3810950 SNP showed strong trends toward significance in acceleration ML and velocity ML. Both parameters were higher in individuals carrying the minor allele, and showed a linear dose dependency. Higher acceleration ML has previously been demonstrated in high-risk individuals for PD (Maetzler et al., 2012) and in clinically overt PD patients (Palmerini et al., 2011), as well as in elderly individuals who are prone to falls (Melzer et al., 2010; Merlo et al., 2012). All these previous studies, in conjunction with our current results, give further support for the proposed influence of the cholinergic system on postural stability. Furthermore, our results suggest: (i) that postural stability, at least in advanced age, is partly determined by genetic predisposition, and (ii) that acceleration and velocity parameters extracted from a challenging quiet standing task are promising markers to describe such (subtle) postural control deficits.

To test for a summation effect, the two above-mentioned SNPs (rs6542746 and rs3810950) were compared simultaneously with postural control parameters. The analysis showed a strong and linear negative trend between number of overall minor alleles and MPF value. In our view, this result also supports the idea of a relevant influence of the cholinergic system on MPF and, in line with this relevant influence, for a particularly high potential of MPF to serve as a “window” into a better understanding of the cholinergic system and its association with human postural control.

The SNP rs1803274 in the *BCHE* gene and the SNP rs2236196 in the *CHRNA4* gene showed no statistically significant associations to postural control parameters in our cohort of healthy older adults. We included these gene variants in this study, as our study was designed as an exploratory analysis and it was intriguing to speculate that the respective gene products could influence postural control due to their relevance within the cholinergic system. However, direct evidence for this hypothesis has not been published, yet, for neither of these variants. Moreover, we investigated a different SNP in CHRNA4 (rs1044396) as mentioned in the literature, which is not in strong linkage disequilibrium. This fact might explain the lack of association of the actual SNP with postural control parameters. This hypothesis has to be tested in future studies. Overall, the influence of the two latter gene variants may be more relevant for the cholinergic system associated with cognitive impairment.

The study faces the limitation that the number of people in the respective genotype groups – except for the SNP in *SLC5A7* – varied greatly with on average 255 individuals being homozygous for the major allele and about 30 individuals for the minor allele. We may thus have overlooked existing influences of some genetic variants due to lack of power. Moreover, with the experimental approach presented here we cannot exclude that our results may be explained -at least partly- by changes occurring at the neuromuscular junction.

## Conclusion

This pilot investigation provides novel information about the association between genetic variants of the cholinergic system and postural control parameters in older adults. Our results should motivate further investigations with larger cohorts and longitudinal study designs.

## Author Contributions

CA, CS, and WM conceptualized the study, analyzed the data, and revised the manuscript. MM drafted the manuscript, was involved in the analysis and interpretation of the data, and provided substantial input to the revisions. US, LZ, FM, TG, GE, A-KH, and DB were involved in data acquisition, and analysis of raw data, and revised the manuscript.

## Conflict of Interest Statement

The authors declare that the research was conducted in the absence of any commercial or financial relationships that could be construed as a potential conflict of interest.

## Footnotes

1 http://www.trend-studie.de
