# Supplementary material for: Cholinergic Pathway SNPs and Postural Control in 477 Older Adults
Source: Front Aging Neurosci. 2018 Sep 4;10:260. doi: 10.3389/fnagi.2018.00260 (PMC6131592; doi:10.3389/fnagi.2018.00260)
Supplement: Supplementary file 1 [file Table_1.docx]

|  |  | **R^2   (with SNP and covariate)^** | **R^2  (without SNP, with covariates)^** | **Effect size (Cohen's f^2^)** |
| --- | --- | --- | --- | --- |
| CHAT | Velocity ML | 0.018 | 0.006 | 0.012 |
|  | Acc ML | 0.018 | 0.006 | 0.012 |
|  | Jerk AP | 0.033 | 0.024 | 0.009 |
|  | Velocity AP | 0.041 | 0.034 | 0.007 |
| SLC5A7 | MPF | 0.143 | 0.119 | 0.028 |
|  | Acc AP | 0.109 | 0.094 | 0.017 |
| SLC5A7+ | MPF | 0.134 | 0.118 | 0.018 |
| CHAT |  |  |  |  |

**Supplemental Table 1: Coefficients of correlation and effect sizes of respective SNPs of the cholinergic system concerning postural control parameters**

Acc = acceleration; AP = anteroposterior; ML = mediolateral; MPF = mean power frequency; SNP = single nucleotide polymorphism;
