# Supplementary material for: Cholinergic Pathway SNPs and Postural Control in 477 Older Adults
Source: Front Aging Neurosci. 2018 Sep 4;10:260. doi: 10.3389/fnagi.2018.00260 (PMC6131592; doi:10.3389/fnagi.2018.00260)
Supplement: Supplementary file 2 [file Table_2.docx]

**Supplemental Table 2: Minor allele frequency of the SNPs included in this study**

| **SNP** | **CHR** | **position**  **(build 37)** | **Gene** | **minor allele** | **major allele** | **MAF** | **MAF 1kg** |
| --- | --- | --- | --- | --- | --- | --- | --- |
| rs6542746 | 2 | 108605836 | *SLC5A7* | C | T | 0.470 | 0.485 |
| rs1803274 | 3 | 165491280 | *BCHE* | T | C | 0.200 | 0.186 |
| rs3810950 | 10 | 50824619 | *CHAT* | A | G | 0.244 | 0.232 |
| rs2236196 | 20 | 61977556 | *CHRNA4* | G | A | 0.270 | 0.269 |

CHR = chromosome; MAF = minor allele frequency; MAF 1kg = allele frequency corresponding to the minor allele extracted from the 1000 Genome project data (only European populations) ([1](#_ENREF_1))

1. Genomes Project C, Auton A, Brooks LD, Durbin RM, Garrison EP, Kang HM, et al. A global reference for human genetic variation. Nature. 2015;526(7571):68-74.
